# «Suspects» in Etiology of Endemic Nephropathy: Aristolochic Acid *versus* Mycotoxins

**DOI:** 10.3390/toxins2061414

**Published:** 2010-06-11

**Authors:** Stjepan Pepeljnjak, Maja Šegvić Klarić

**Affiliations:** Department of Microbiology, Faculty of Pharmacy and Biochemistry, University of Zagreb, Schrottova 39, HR-10000 Zagreb, Croatia

**Keywords:** endemic nephropathy, aristolochic acid, ochratoxin A, citrinin, DNA-adducts

## Abstract

Despite many hypotheses that have been challenged, the etiology of endemic nephropathy (EN) is still unknown. At present, the implications of aristolochic acid (AA) and mycotoxins (ochratoxin A—OTA and citrinin—CIT) are under debate. AA-theory is based on renal pathohistological similarities between Chinese herbs nephropathy (CHN) and EN, findings of AA-DNA adducts in EN and in patients with urinary tract tumors (UTT), as well as the domination of A:T®T:A transversions in the p53 mutational spectrum of UTT patients, which corresponds with findings of such mutations in AA-treated rats. However, exposure pathways of EN residents to AA are unclear. Experimental studies attempting to deduce whether nephrotoxins OTA and CIT appear at higher frequencies or levels (or both) in the food and blood or urine of EN residents support the mycotoxin theory. Also, some molecular studies revealed the presence of OTA-DNA adducts in the renal tissue of EN and UTT patients. In this review, data supporting or arguing against AA and mycotoxin theory are presented and discussed.

## 1. Introduction

Endemic nephropathy (EN) is a chronic kidney disease that affects the human population of some rural areas in Bosnia and Herzegovina, Bulgaria, Croatia, Romania, and Serbia. In Croatia, the EN area is restricted to 14 villages in the western part of Brodsko-Posavska County. The EN was first described in the late 1950s, and an association between the disease and urinary tract tumors (UTT) was recognized in the 1970s. This fatal disease is characterized by a focused and restricted geographical distribution, occurrence in farming households, high mortality from uremia, and high incidence of UTT. The endemic nature of the disease implicates the involvement of environmental factors, particularly of naturally occurring toxins and carcinogens. In the past 50 years, many studies exploring the possible role of various environmental factors in EN and UTT have been performed, and this includes research on heavy metals and minerals, bacteria (β-haemolytic streptococci, *E. coli*, and *Leptospira* spp.) and viruses (coronaviruses), Pliocene lignites, aristolochic acid (AA), and mycotoxins (particularly ochratoxin A, OTA) [1,2,3,4,5]. 

Aristolochic acid is a generic name for family of nitrophenantrene derivatives that can be found in the stem and seeds of *Aristolochia* species, which occur in flooded areas in many parts of the world, including EN areas. Between 1990 and 1992, a number of cases of interstitial nephropathy were reported in young women in Belgium who were undergoing a slimming regimen with Chinese herbs. Chemical analysis of these Chinese herbs remedies did not reveal nephrotoxic contaminants of fungal or plant origin, such as ochratoxin A or AA [6]. Vanhaelen *et al*. [7] used a different analytical procedure for AA detection, but again, AA was never found in these weight-loss pills. In another study, the same group of investigators detected AA, instead of tetrandrine, in 10 out of 12 herbal powders in Belgium pharmacies sold between 1990 and 1992 under the name *S. tetrandra*. However, these findings are not related to the reported renal failure in women involved within the weight-loss program. It seems that other chemicals, rather than AA alone, might be involved in producing the nephrotoxic and carcinogenic effects related to the weight-loss pills [8]. Nortier *et al*. [9] found high level of AA-related DNA adducts in renal specimens obtained from the Chinese herbs nephropathy (CHN) patients (38/39) involved in weight-loss program, which supported chronic exposure to this phytotoxin. The authors also detected lower levels of OTA-related DNA adducts in four out of 25 samples, and concluded that OTA does not have a key role in the nephropathy of these CHN patients. Cosyns *et al*. [10] tested the nephrotoxic effects of pure AA and slimming regimen + AA on rats. At a dose of 10 mg/kg *per os* 3 months, no renal fibrosis was detected, but tumors of the renal pelvis, urinary bladder, and forestomach were found in rats. In addition, 2/4 rats treated with the slimming regimen + AA developed tubulointestitial fibrosis, which suggested that the toxicity of AA could be potentiated. Some clinical and morphological features of CHN (anemia, proteinuria, renal athrophy, interstitial fibrosis, and urothelial malignancy) are similar to those observed in EN patients [11,12]. About 35 years ago, Ivić [13] observed that the seeds of birthwort *A. clematitis* (vučja stopa in Croatian) were sometimes co-mingled with wheat grain, and suggested that exposure of EN patients to AA could occur through contamination of flour and baked bread. 

In the 1970s, similarities between EN and ochratoxin A (OTA)-induced nephropathy in pigs were observed, and it was suggested that this mycotoxin could be involved in the etiology of EN. Since then, the nephrotoxic and carcinogenic properties of OTA have been demonstrated on a number of experimental animals including pigs, rats, mice, or poultry [1,2,3,4,14]. In the past few decades, many surveys in EN areas have confirmed the presence of OTA in a variety of diet products, including cereals and smoked meat [15,16]. It was also found that people in EN regions are often exposed to higher concentrations of OTA, which was confirmed by the presence of higher levels of this toxin in the blood and urine of subjects from EN regions, as compared to those living in EN-unaffected areas [17,18,19]. 

According to current research data of Grollman *et al.* [20], AA is a “prime suspect” in the etiology of EN, but the role of mycotoxins in the development of this disease could not be excluded. This review is aimed at discussing the hypotheses on the implication of AA and mycotoxins in the etiology of EN.

## 2. Epidemiological and Clinical Features of EN and CHN

In the period of 1991–2002, the average general mortality in the EN region of Croatia was 10.3/1000, while the specific mortality for EN patients was 0.58/1000 (men) and 0.72/1000 (women). The average age of death of EN patients was 67.7 (men) and 70.3 (women), which is significantly higher than in the sixties (45.1 years). Between 1995 and 2002, the specific mortality from UTT in Croatian EN region (6.902/100000) was 55-times higher than in the entirety of Croatia (0.126/100000). Similar findings were reported for EN region of Bulgaria [3,21].

The epidemiological and clinical picture of EN and CHN has been recently reviewed [22]. The clinical features and pathomorphological changes of EN encompass tubular degeneration, interstitial fibrosis, hyalinization of glomeruli, enzymuria, loss of weight, pale skin, and absence of hypertension. Characteristic biochemical changes include mild proteinuria, glucosuria, mononuclear cell infiltration, increased blood urea nitrogen concentration, creatinine and urinary enzymes (γ-glutamyltransferase, alkaline phosphatase, lactate dehydrogenase), increased urinary pH, anemia, and increased IgM and IgG levels. 

Several clinical signs could be observed in both EN and CHN patients, such as tubular functional abnormalities, intersititial fibrosis, frequent malignancies of the urothelial tract (40%), normal arterial blood pressure, increased serum creatinine levels, mild tubular proteinuria, normoglycemic glucosuria, and anemia. The renal biopsies of EN and CHN patients revealed similar findings, such as hypocellular interstitial sclerosis and tubular atrophy with normal or sclerosed glomeruli, depending on the stage of the disease [11,12,22]. 

Despite some similarities, there are also some striking differences between EN and CHN that are manifested in some epidemiological features and in the course of the disease. EN affects only rural populations (aged 45–60) in restricted areas of Bosnia and Herzegovina, Bulgaria, Croatia, Romania, and Serbia, while CHN was observed as an epidemic in both urban and rural populations (aged 35–45) in Belgium (exclusively women), as well as in other European countries and the USA. Familiar clustering is very important for EN, but not for CHN. Migrants develop EN after one or more decades of residence in EN regions, with a sex ratio of 1:1. In contrast, CHN is usually developed rapidly (6 to 24 months of exposure), and until now, was dominant in young women exposed to a slimming regimen containing AA. In addition, UTT development is very slow; it peaks one decade after the peak of EN, while the malignant progression of CHN takes two to six years. The other differences include a lack of uterohydronephrosis in EN, except in the cases of UTT, and less prominent involvement of the columns of Bertin in autopsied EN than in CHN patients [11,12,21,22]. In addition, endemic nephropathy in Tunisia and karyomegalic interstitial nephropathy has been associated with OTA. Both diseases share clinical and pathological similarities with EN [23]. 

## 3. Evidences for the Implication of AA and Mycotoxins in EN

### 3.1. Field exposure studies

So far, there is no evidence that people in EN areas are exposed to AA through food consumption, particularly of bread made from homegrown wheat. Recently, Hranjec *et al.* [13] attempted to find, by means of questionnaire, whether EN patients were more exposed to AA than the healthy residents of EN villages in Croatia. The majority of subjects, including 90% of EN patients, recalled that *A. clematitis* was frequently found in the fields 20–30 years ago. Since then, all studied groups confirmed a significant increase in the use of herbicides, as well as a reduction of *A. clematitis* in the fields. However, there is no evidence to confirm that AA contamination of homemade bread occurred, or that the geographical pattern of AA exposure is consistent with occurrence of EN. In July of 2007, we conducted a similar questionnaire (unpublished data) among residents (N = 40), aged 20–80 years, in four EN villages (Lužani, Živike, Pričac, Slavonski Kobaš). Our questionnaire was designed to explore the subjects’ dietary habits and to find out if potential exposure to AA could occur through wheat flour contamination (Table 1). 

**Table 1:** Questionnaire conducted in July of 2007 among residents (N = 40) of four villages (Lužani, Živike, Pričac, Slavonski Kobaš) in the EN region of Croatia.

Questions	Yes	No
EN patients	2.5% (man, age 58)	97.5%
UTT patients	2.5% (women, age 50)	97.5%
EN family history	22.5%	77.5%
*Sighting of *A. clematitis* in fields	77.5% (particularly in meadows)	22.5%
Consummation of *A. clematitis* as a part of remedies	-	100%
Unknown origin of flour for homemade bread	100%	-
Consummation of smoked meat products	60% on daily basis, 40% periodically	-

* Residents were shown the photo of *A. clematitis* and a specimen in the herbarium.

The most important information collected is that the wheat was harvested in the mid of July, when *A. clematitis* is still immature. These observations are supported by the findings of Long and Voice [24]. The authors also indicated a possibility of ingestion of *A. clematitis* as a medicinal herb. According to the results of our questionnaire, EN residents never used this plant as a regimen. The second important fact is that both 20–30 years ago, and today, residents of EN and non-EN villages in Brodsko-Posavska County brought their wheat to mill and exchanged it for sacks of flour of unknown origin. Taking into account these observations, it seems that human exposure to AA through wheat flour contamination is unlikely. Besides that, people in these regions frequently consume homemade smoked meat that is often contaminated by molds, as it was previously reported by Pepeljnjak and Blažević [25]. Some elderly residents believe that mold cover on smoked meat provides a good protection from flies and should not be removed. Also, some residents (16%) recalled that 20–30 years ago, moldy corn was used for the preparation of homemade brandy. These observations only partially illustrate the specific dietary habits that suggest higher exposure of EN residents to mycotoxins. 

The occurrence of OTA alone and the co-occurrence of OTA with other nephrotoxic mycotoxins in food and biological samples from EN and non-EN regions are summarized in Table 2 and Table 3. Higher contamination of both OTA and citrinin (CIT), or both OTA and fumonisin B_1_ (FB_1_), were found in EN, as compared to non-EN, villages in Bulgaria, Croatia, and Serbia. Exposure of EN residents to these mycotoxins was confirmed by the identification of both OTA and CIT in blood and urine, and by higher sphinganine/sphingosine ratios in urine (biomarkers of exposure to fumonisins) [26,27,28,29,30,31,32,33,34,35,36]. Studies in the non-EN regions of Croatia, and in other European countries, show that people are also exposed to a variable amount of OTA [18]. The estimated daily intake of OTA in non-EN regions of Croatia (0.4 ng/kg b.w.) is lower than the tolerable daily intake proposed by the World Health Organization (16 ng/kg b.w.) [19]. However, studies conducted in EN areas of Croatia and Bulgaria confirmed that the EN population is more frequently exposed to OTA, and possibly to other nephrotoxic mycotoxins, because of microclimatic conditions (high humidity) and specific dietary habits. Recently, OTA and CIT, but not AA, were detected in the food and urine of the members of three EN families, which confirmed dietary exposure of the EN population to mycotoxins, but not exposure to AA [33]. Analysis of weekly collected urine showed that 0.1 to 0.43 μg/L of OTA was present in 56% of the samples, while 0.37 to 2 μg/L of CIT was found in 9% of urines. Both toxins were detected in 6% of the samples. In addition, OTA was detected in 42% of the blood samples of EN patients, and in the blood of three karyomegalic nephropathy patients in Tunisia in high concentrations ranging from 1 to 1136 ng/mL, and from 102.63 to 1023 ng/mL, respectively [23].

**Table 2:** Occurrence of some nephrotoxic mycotoxins in food from EN and non-EN regions.

Samples	Country (year)	Mycotoxin	EN		non-EN		
			%	range (μg/kg)	%	Range (μg/kg)	Ref.
Smoked meat	Croatia (1978–80)	OTA	12–29	10–920	not analyzed		[25]
Cereals	Croatia (1979–81)	OTA	45	10–68900	15	230–4700	[16]
Beans	Bulgaria (1989–90)	OTA	36.6–40	25–260	5–8	10–220	[26,27]
		CIT	36–40	30–800	10–12	20–200	
Corn	Bulgaria (1989–90)	OTA	~44	25–900	5–8	10–235	[26,27]
		CIT	40–43	50–1100	10–12	50–380	
Corn	Croatia (1996–97)	OTA	9–50	0.29–613.7	10–20	0.26–223.6	[28]
		FB_1_	~96	12–11661	90.7–98	12–11278	
Cereals	Bulgaria (1999)	OTA	35	<0.5–140	35	0.65–1.9	[29]
		CIT	9.4	<5–420	5	<5–6.5	
Cereals	Croatia (1999–00)	OTA	81.8–100	0.019–160	11.1–88.9	0.019–32.3	[30]
Beans	Croatia (2001)	OTA	not analyzed		37.7	0.25–0.92	[31]
Corn	Croatia (2002)	OTA	not analyzed		33.3	0.73–2.54	[32]
		FB_1_			100	196.8–1377.6	
		FB_2_			13	68.4–3084	
Cereals	Croatia (2007)	OTA	16.2	2.5–31.7	not analyzed		[33]
		FB_1_ + FB_2_ + FB_3_	27	200–20700			

**Table 3:** Occurrence of OTA in biological samples from EN and non-EN regions.

		EN		non-EN		
Samples	Country (year)	%	range	%	range	Ref.
Pigs blood	Croatia 1979/81	0–15.7	0–37 μg/mL	not detected		[10]
Pigs liver	Croatia 1979/81	0–10.5	0–21 μg/kg	not detected		
Pigs kidney	Croatia 1979/81	5.2	16–27 μg/kg	not detected		
Human blood	Croatia 1985–94	0.2–4.5	2–50 ng/mL	0.4–2.4	1–10 ng/mL	[12]
Human blood	Croatia 1997/98	not analyzed		59	0.21–15.9 ng/mL	[13,15]
Human blood	Bulgaria 2002/03	100	0.1–10.9 μg/L	not analyzed		[31]
Human urine	Bulgaria 2002/03	88–97%	0.01–1.91 μg/L	not analyzed		

### 3.2. Experimental studies

Studies on experimental animals using AA and mycotoxins showed both similarities and differences with EN in humans. 

After 17 to 21 months of intraperitoneal administration of 0.1 mg AA/kg b.w. to New Zealand White rabbit females, all animals displayed features that are common to both CHN and EN: intestitial fibrosis, increase in serum creatinine, glucosuria, proteinuria, and anemia, and three out of 12 developed urothelial tumors. Additionally, fibrotic changes were also observed in the stomach, with tumors in the peritoneal cavity [36]. Mice and rats were found to be less susceptible to AA; mice developed forestomach cancer after administration of 5 mg/kg b.w. for 56 weeks, and rats after 1 or 10 mg/kg b.w. for three months [38]. Rats developed renal pelvis and urinary bladder carcinoma after oral treatment with a relatively high dose of 10 mg AA/kg per day for five days [10,11].

Nephropathy in pigs taking feed that contained 0.2, 1, and 4 mg OTA/kg showed similarities with EN in humans, including major lesions in proximal tubules, followed by an increase of glucose and protein urinary excretion, elevated serum urea and creatinine levels, and an increase of urinary enzymes activities [39]. Pfohl-Leszkowicz and Manderville [21] reviewed OTA carcinogenicity studies that confirmed OTA carcinogenic potency in rodents. B6C3F1 mice and Fischer rats developed renal tubular adenomas and carcinomas after 1 and 2 years of repeated, low dosage of OTA (40 μg in the diet and 21, 70, 210 μg/kg b.w. administrated intraperitoneally, respectively). At high doses, all rats presented karyomegalies. Male rodents were more susceptible to OTA carcinogenicity than females: 72% of male rats and 16% of female rats developed renal carcinomas, while none of mice females had renal carcinoma or adenoma. Also, OTA induced adenocarcinoma and transitional cell carcinoma of the bladder in male rats only [40]. These sex-related differences have been recently explained by OTA binding to α2u-globulin, which is a carrier specific only to male rats. OTA is delivered to the proximal tubule epithelia, and thus cell exposure to the mycotoxin is increased in males, but not in female rats. There are no analogous androgen globulins in humans [41]. Also, male pigs, as well as male rats, showed different expressions of biotransforming enzyme in the kidneys than what females showed [42,43]. It is also important to note that EN tumors occur in the transitional cell tissues of the urinary tract, and are not renal cell carcinomas. However, extrapolation from the urological carcinogenesis in experimental animals to human urinary tract tumors is especially difficult. 

Besides OTA, CIT and FB_1_ could also play an important role in development of EN, as well as other chronic kidney diseases of unknown etiology. This hypothesis is supported by the fact that these mycotoxins have synergistic, or at least additive interactions *in vitro* and *in vivo*: (1) the combination of OTA and CIT was found to have additive or synergistic effects on kidney impairment in chicks, mices, rats, guinea pigs, dogs, and swines, and synergistic cytotoxic effects on porcine and human kidney cell lines [44,45,46]; (2) the cytotoxic synergism of OTA and FB_1_ was reported for green monkey kidney Vero cells, rat brain glioma cells, and human intestinal Caco-2 cells [47]; (3) the combined treatment of porcine kidney PK15 cells with OTA and FB_1_applied in low doses resulted in dominant pro-oxidative and genotoxic additive interactions and synergistic apoptotic effects [48,49,50]; and (4) the combined FB_1_ and OTA treatment of rats resulted in synergistic oxidative DNA damage of the kidney cells and an increased rate of OTA-DNA adduct at doses that correspond to daily human exposure in Europe [51,52].

### 3.3. Bioactivation of AA and OTA and molecular studies

A few enzymes and coenzymes are implicated in AA bioactivation, including hepatic cytochrome P450 enzymes (CYP1A1, CYP1A2) and NAD(P)H: quinone reductase (NQO1), as well as renal NQO1, cytochrome P450 reductase (CPR) ,cyclooxygenase (COX), and sulphotransferase (SULT1A1). These enzymes are involved in biotransformation of AA to *N*-hydroxyaristolactam I, which can be oxidized by renal peroxidases to form persistent adducts with DNA. These adducts are known to be mutagenic and can initiate tumors [53,54]. dA-aristolactam (AL) and dG-AL DNA adducts have been identified in the renal cortex of EN and UTT patients (from 0.6 to 1.6 adducts/10^8^ nucleotides) but not in patients with other chronic kidney diseases [20]. It was also found that EN patients homozygous for the NQO1*2 allele were at increased risk of developing UTT (OR 13.75), suggesting the importance of NQO1 in AA activation and development of nephropathy [55,56]. An analysis of p53 mutations in AA-treated rats showed that predominant mutation was an A:T®T:A transversion [20]. Such a mutation, which accounted for 78% of all base substitutions, was also found in EN-associated UTT patients from Croatia and Bosnia, which supports the implication of AA in EN [20,57]. In contrast, in a study of blood samples of 90 Bulgarian EN patients, these p53 mutations were found only in 10% of the samples [58]. Pfohl-Leszkowicz [34,52] pointed out several reasons why the implication of AA in both CHN and EN is doubtful: (i) findings of AA-DNA adducts in Belgian women suffering from CHN [58] after intake of slimming pills in which AA was not detected [34]; (ii) detection of AA-DNA adducts at high levels (five adducts/10^7^ nucleotides) several years after the slimming regimen was stopped [59]; (iii) analysis of DNA samples of women taking slimming pills revealed OTA-DNA adducts, but no AA-DNA adducts [34]; (iv) AA-DNA adducts detected with Polyacrylamide Gel Electrophoresis (PAGE) that were reported by Grollman *et al.* [20] in EN and UTT patients was found questionable due to the low purity of DNA after extraction, as well as an unclear standard of comparison for AA-DNA adduct bands with those within the sample. However, AA-DNA adducts found in EN and UTT patients were confirmed by liquid chromatography electrospray ionization/multistage mass spectrometry (LC-ESI/MS/MS) techniques [20]. Nevertheless, OTA-related DNA adducts have not been analyzed in these EN patients and the occurrence of AA or AA-metabolites in food or urine samples from the EN region has not been tested. 

OTA genotoxic action is still under debate. Based on the current literature, OTA genotoxicity may be assigned as direct (DNA adduct formation) and indirect (oxidative DNA damage) OTA mechanisms of action [21]. The experiments with microsomes from transgenic mice showed that the formation of OTA-mediated DNA adducts (analyzed by ^32^P-postlabeling method) are under the control of biotransformation enzymes, such as CYP450 1B1, 2C9, COX, and LOX [21]. A study on male Dark Agouti rats showed that the genotoxicity of OTA is related to CYP2C11 expression (corresponding to human CYP2C9), which is able to metabolize debrisoquine [42]. The roles of genetic polymorphisms in genes of some detoxifying and conjugation enzymes have been studied in EN patients. It was found that the CYP3A5*1 allele, as a marker for CYP3A5 expression in the human kidney, is associated with increased risk for EN (OR 2.41) [60]. It is possible that carriers of the CYP3A5*1 can more efficiently convert OTA into genotoxic metabolites [52]. Lebrun *et al.* [61] found an association between polymorphisms in GST conjugation isoenzymes and DNA damage induced by OTA. A mechanism by which OTA acts as a genotoxin has been proposed [21]. The hydroquinone metabolite OTHQ covalently binds to DNA by an autoxidation or OTA *in vitro* activation in pig kidney microsomes or in human kidney cells. Additionally, OTA facilitates C8 deoxyguanosine adduct (O-C8-dG-OTA and C-C8-dG-OTA) formation in pigs, rodents, and human kidney cells, one of which is also formed when COX and LOX enzymes are highly expressed. It was shown that *in vitro* induction of LOX and COX2 favored the formation of C-C8-dGMP-OTA, whereas CYP2C9 and COX1 favored the formation of the OTHQ-related DNA adduct [34]. Recently, the C-C8dGMP-OTA adduct has been identified by liquid chromatography-tandem mass spectrometry (LC-MS/MS) in the kidneys of Dark Agouti and Fischer rats (20–70 adducts/10^9^ nucleotides) treated with approximately 8.3 and 6.8 mg of OTA/kg b.w. for three days, respectively [62]. These adducts were also detected in EN patients from Bulgaria, Croatia, and Serbia, as well as in French patients with kidney carcinoma (1–115 per 10^9^ nucleotides), but not in non-EN patients [34]. Despite the evidence for the genotoxicity of OTA, other researchers could not detect OTA-related DNA adducts via the method of using radiolabeled OTA in combination with liquid scintillation counting or accelerator mass spectrometry, or by 32P-postlabeling and LC-MS/MS techniques [63]. Instead of direct OTA genotoxicity, they favor an indirect mechanism that involves oxidative stress, which is supported by OTA-mediated lipid peroxidation, formation of ROS, depletion of glutathione, and oxidative damage of DNA *in vitro* and *in vivo* [64]. These controversies have been explained by differences in the DNA extraction and ^32^P-postlabeling methodology [21,52]. In addition, citrinin (CIT) generates DNA adducts in human kidney cells, which was also detected in the kidneys of EN patients. Nevertheless, AA-DNA adducts were not observed in these subjects [34]. Also, FB_1_ enhances OTA-DNA adduct formation in pigs and rats, and the DNA adduct pattern is similar to that found in some EN patients in Croatia [52]. 

**Table 4:** Evaluation of evidence for the “prime suspects” in the etiology of EN and UTT.

Evidences	AA	*versus*	OTA
Nephrotoxic	+		+
Carcinogenic	+		+
Clinical picture similarities with EN-UTT	+/-		+/-
DNA-adducts	+?		+
Field exposure evidences (food, urine, blood)	-		+
Higher exposure in EN over non-EN	-		+/-
Toxic levels	lack of evid.		+

+ = yes; - = no; ? = under debate.

## 4. Conclusions

The etiology of EN and associated UTT is still an open question. Based on epidemiological features of EN, researchers agree that the causative agent of both diseases is of natural origin. To resolve this “mystery,” certain criteria must be included in the evaluation process: (1) studies that will confirm exposure to the suspected agent in the EN area and evidence that the levels of exposure to this agent are higher than in non-EN areas; (2) toxicological studies on the biomarkers of exposure and the biological effects that will associate this agent with EN and UTT in humans. With respect to these criteria, the presented data (Table 4) pleads for the implication of OTA in the etiology of EN. Based on toxicity and carcinogenicity studies, OTA is a nephrotoxin and a carcinogen, but the mechanism of carcinogenicity remains unclear. Also, a consensus on appropriate risk metrics for OTA is lacking. Recently, Kuiper-Goodman *et al.* [65] re-evaluated risk metrics for OTA and showed that the two approaches converge whether one assumes a threshold mode of action (pig study [38] and modified uncertainty factors), or a non-threshold mode of action (rat carcinogenicity study reviewed in [21]), with both leading to a tolerable daily intake (TDI) or negligible cancer risk intake (NCRI) in the order of 4 ng kg b.w.^-1^ per day. Taking into account the amounts of OTA found in food, often toxic levels are found, at least considering cancer development. Although OTA was detected in the food and in the blood of humans from non-EN parts of the world, this fact does not discard the hypothesis of the role of OTA in the development of EN because the population in EN region is often exposed to a higher amount of this toxin than in non-EN areas. Animals treated with AA or OTA displayed some features that are common to EN and UTT; although, there are some discrepancies, such as forestomach cancer in animals exposed to AA, or sex-related renal and UTT carcinomas in those treated with OTA. However, extrapolation from carcinogenesis in experimental animals to humans is especially difficult. The DNA-AA and DNA-OTA adducts that have been reported in EN patients and experimental animals are currently under debate, mainly due to DNA extraction and ^32^P-postlabeling methodology. Nevertheless, OTA-DNA adducts have been repeatedly reported in EN patients and the structure of one adduct has been recently confirmed by LC-MS/MS [62]. The route of exposure to AA is doubtful and should be determined. Since AA undergoes extensive metabolic processing, the exposure of humans to this agent should be evaluated by the measurement of AA-metabolites in fluids, as well as by the measurement of pure AA in food. Genotoxic action of OTA and the presence of several OTA metabolites in the blood and urine of EN patients observed by Pfohl-Leszkowicz *et al.* are solid arguments in favor of the hypothesis of OTA implication in EN and associated UTT development. More attention should be focused on molecular studies that can reveal a possible involvement of predisposing factors, such as the alleles for detoxifying and conjugation enzymes, since these proteins are involved in the activation of many natural toxins. In addition, surveys on the simultaneous occurrence of nephrotoxic mycotoxins, as well as biomarkers of exposure to such agents, should be further explored in both the EN and non-EN regions, since most of the conducted biological investigations confirmed their synergistic, or at least additive toxic interactions. 
